# Different Patterns of Neural Activity Characterize Motor Skill Performance During Acquisition and Retention

**DOI:** 10.3389/fnhum.2022.900405

**Published:** 2022-06-13

**Authors:** Dorsa Beroukhim-Kay, Bokkyu Kim, John Monterosso, Rebecca Lewthwaite, Carolee Winstein

**Affiliations:** ^1^Motor Behavior and Neurorehabilitation Laboratory, Division of Biokinesiology and Physical Therapy, Herman Ostrow School of Dentistry, University of Southern California, Los Angeles, CA, United States; ^2^Neuroscience Graduate Program, University of Southern California, Los Angeles, CA, United States; ^3^Southern California Clinical and Translational Science Institute, University of Southern California, Los Angeles, CA, United States; ^4^SUNY Upstate Medical University, Syracuse, NY, United States; ^5^Department of Psychology, University of Southern California, Los Angeles, CA, United States; ^6^Division of Biokinesiology and Physical Therapy, Herman Ostrow School of Dentistry, University of Southern California, Los Angeles, CA, United States; ^7^Rehabilitation Therapies Division, Rancho Los Amigos National Rehabilitation Center, Downey, CA, United States; ^8^Department of Neurology, Keck School of Medicine, University of Southern California, Los Angeles, CA, United States

**Keywords:** motor learning, neural mechanisms, skill acquisition, skill retention, fMRI, neuroplasticity

## Abstract

Motor performance and learning have distinct behavioral and neural signatures and can be uniquely modulated by various informational and motivational factors. Contemporary frameworks describe four different motor learning mechanisms mapped onto specific neural regions which are key for motor skill acquisition: error-based learning (cerebellum), reinforcement learning (basal ganglia), cognitive strategies (prefrontal cortex), and use-dependent learning (motor cortex). However, little is known about the neural circuits engaged during skill acquisition that are modulated specifically by practice-based performance improvement and those that predict recall performance. Based on previous work, we hypothesize that brain activity during practice in primary motor cortex and basal ganglia (1) is associated with trial-by-trial practice performance and (2) is predictive of immediate recall performance. Leveraging the contemporary framework, we use a well-known task paradigm that primarily relies upon cognitive strategy, reinforcement, and use-based learning mechanisms to test our hypotheses. Forty neurotypical young adults were asked to practice a pinch force tracking task. Participants received performance feedback after each trial during practice. We used whole brain analysis of functional magnetic resonance imaging (fMRI) and behavioral performance measures (i.e., time-on-target and self-efficacy) during the practice phase to determine which brain activation patterns are (1) associated with trial-by-trial tracking performance and (2) predictive of immediate no-feedback retention performance. We observed brain activations in the frontal orbital cortex, putamen, amygdala, and insula correlated with tracking performance improvement during practice. In contrast, a different set of performance-related activated regions were observed that were associated with immediate retention performance that included the primary motor cortex, superior frontal gyrus, somatosensory cortex, angular gyrus, and parietal gyrus. Our findings demonstrate that improved practice performance and recall of a sensorimotor skill are correlated with distinct neural activity patterns during acquisition, drawing on different motor learning mechanisms during encoding. While motor performance improvements depend on both cortical and subcortical regions, motor skill recall depends primarily on prefrontal and motor cortices. We discuss possible interpretations for why our hypothesis regarding basal ganglia activity and retention performance was not supported. Understanding the different neural mechanisms engaged in motor performance and learning may inform novel interventions to enhance motor skill learning.

## Introduction

The ability to acquire and retain motor skills is important in various developmental, occupational, and rehabilitative settings. The neural substrates that mediate this ability are thought to depend primarily on specific learning mechanisms (Krakauer et al., 2019). Contemporary frameworks describe four major motor learning mechanisms, including error-based learning, reinforcement learning, cognitive strategies, and use-dependent learning, that contribute to motor performance and skill retention (Krakauer et al., 2019; Spampinato and Celnik, 2021). The specific task demands and stage of skill acquisition elicit one or more of these motor learning mechanisms including the associated neural substrates.

For example, there is a body of work indicating the role of the cerebellum in early stages of motor acquisition, that accounts for systematic changes in error reduction on a trial-by-trial basis (error-based learning) (Galea et al., 2007; Jayaram et al., 2011; Cantarero et al., 2015; Spampinato and Celnik, 2017). In addition, both animal and human studies provide evidence that changes in motor cortex excitability (use-dependent learning), prefrontal cortex involvement (cognitive strategies) and basal ganglia input (reinforcement learning) contribute to encoding of the motor skill later in the acquisition period (Stefan et al., 2006; Rosenkranz et al., 2007; Dayan and Cohen, 2011; Kantak and Winstein, 2012; Spampinato and Celnik, 2021).

While there have been many investigations of brain activation associated with processes and stages of learning during acquisition (encoding), less attention has been given to examining brain areas activated during practice that specifically correlate with task practice performance and recall performance during retention tests.

Neuroimaging investigations have begun to shed light on brain activity during acquisition that is correlated with practice and retention performance. Specifically, non-invasive brain stimulation studies in humans have recently revealed that primary motor cortex (M1) excitability during practice is proportional to skill retention performance the next day in a sequential visual isometric pinch task (Cantarero et al., 2013; Spampinato and Celnik, 2017). A combined transcranial magnetic stimulation (TMS) and functional magnetic resonance imaging (fMRI) study, showed that increased activation in prefrontal, premotor and parietal areas, in addition to M1 excitability during practice, correlates with retention of a serial reaction time sequence task in humans (Lin et al., 2011). While the involved neural correlates of motor learning mechanisms have been documented, it is important to recognize that these learning processes are also known to be influenced by various motivational factors.

Motivation, classified as either extrinsic or intrinsic can exert a powerful influence on learning and memory behavior. Extrinsic motivation refers to being driven to do something because it leads to an independent outcome, such as working for monetary reward or studying for a good score. Intrinsic motivation, on the other hand refers to an intrinsic source, to being moved to doing something because it is inherently interesting and perhaps satisfies fundamental psychological needs for competence, autonomy, and social relatedness (Ryan and Deci, 2000).

Recent studies are beginning to examine the behavioral impact and mechanistic processes of monetary reward (extrinsic motivation) (Wächter et al., 2009; Abe et al., 2011; Steel et al., 2016) in combination with and distinguished from the effect of performance feedback alone on motor learning (Widmer et al., 2016; Codol et al., 2020; Sporn et al., 2021; Vassiliadis et al., 2021). These studies provide evidence for the role of the basal ganglia in modulating the beneficial effect of extrinsic motivation on motor performance. Less has been done to examine the neural influence of intrinsic motivational factors, such as enhanced expectations or self-efficacy (which support the psychological need for competence) on motor learning mechanisms. In our study, we explored the effects of carefully crafted intrinsic motivation statements in addition to performance feedback by comparing the behavioral and neural effects of these two variables together versus performance feedback alone.

This experiment was designed to fill three related gaps in knowledge described above. We devised a study whereby all participants receive knowledge of results (KR) performance feedback (information) after every trial while half the participants also receive motivational statements (motivational instructions and feedback) during practice of a well-known pinch force tracking task (Abe et al., 2011; Steel et al., 2016; Spampinato and Celnik, 2017). We chose this task paradigm to elicit the four features of the contemporary framework of motor learning processes (error-based learning, reinforcement learning, cognitive strategies, and use-dependent learning). The goal of the task was “to keep the cursor inside the target box.” Therefore, the visual feedback (Knowledge of Performance, KP) provided in real-time reflects the degree to which the cursor is outside of the target box. In essence the KP represents a dynamic error signal that can be used through error-based learning to improve performance (i.e., increase time-on-target). To reduce this error, the participant will need to engage cognitive strategy learning mechanisms in an attempt to compress the force pad in such a way as to maximize the time the cursor is inside the target box. As performance improves through the use of error-based and cognitive strategy learning mechanisms, time-on-target feedback (KR) provided after each trial provides information about the success of achieving the goal; this in turn, elicits a third learning mechanism termed reinforcement learning. Finally, the trial-to-trial practice of the tracking task over 72 repetitions elicits the use-dependent learning mechanism associated with task-specific practice.

Here, we use whole brain analysis of fMRI during practice to address the following two primary aims: to identify neural correlates of motor practice that: (1) are associated with performance improvement during acquisition (encoding) of a sensorimotor tracking task, and (2) predict immediate retention performance (without feedback). The third aim is to explore the impact of intrinsic motivational statements on behavioral and neural correlates of motor skill learning.

Based on the task demands for skill acquisition, our hypotheses for the two primary aims are that: engagement of prefrontal cortex, basal ganglia, and motor cortex will be associated with greater performance improvement during the acquisition phase and specifically we expect that motor cortex and basal ganglia activity during acquisition will be predictive of recall performance at immediate retention. To address our third aim, we curated our motivation statements, which were based on previous behavioral studies and were designed around three criteria: to enhance self-efficacy expectations, induce a positive (growth or incremental) concept of ability mindset (the notion that abilities can be developed through effort and persistence) (Dweck, 1986) and support perceived competence, using positive comments/praise about the participant’s performance (Lewthwaite and Wulf, 2017). We hypothesize that the intrinsic motivational statements will boost performance and be associated with an increase in basal ganglia activity through an enhancement of reinforcement learning processes.

## Materials and Methods

### Participants

Forty-three participants were sequentially recruited into the study. Three individuals were excluded from all analyses because of incomplete behavioral and fMRI data. All three exclusions were due to scheduling conflicts for the neuroimaging scan. 40 subjects [25 female, mean age (SD) = 23.83 (4.63) years] participated (Table 1). All participants were right-hand dominant, able to undergo an MRI and had normal or corrected to normal vision (contact lenses) (self-assessed via eligibility requirements to participate). Hand preference was assessed using the Edinburgh Handedness Inventory (Oldfield, 1971), confirming that all participants were right-hand dominant. Participants were a sample of convenience recruited from the University community and had no prior experience with the motor task. All participants gave informed consent, and the study was performed with University of Southern California (USC) Institutional Review Board approval (HS#-13-00817).

**TABLE 1:** Participant characteristics.

	Overall	Motivation plus feedback group	Feedback only group
*N*	40	20	20
Age (*SD*)	23.8 (4.6)	23.4 (5.4)	24.3 (3.8)
Sex, male/female	15/25	9/11	6/14

*For fMRI analysis, data from 6 participants were removed, leaving n = 18 in motivation plus feedback group and n = 16 in feedback only group.*

### Experimental Design

We used a single group design to determine the neural correlates of motor performance improvement during practice and those that were predictive of retention performance. For the exploratory analysis, we used a between-group design to examine effects of motivational statements plus informational performance feedback (KR, i.e., time on target) compared to performance feedback alone on brain activity and concurrent motor performance.

Participants lay supine in the MRI tube and held a pressure sensor (TSD110-MRI, BioPac) between the thumb and index finger of the right dominant hand. The pressure sensor was connected to an air tube, which led to the MRI control room, where it was connected to a transducer converting air pressure to voltage. Pinching of the pad resulted in generation of a differential voltage signal. The recorded signal was used to provide participants with real time visual feedback of the cursor movement. The cursor movement and moving target presentation were instantaneously displayed on a projector screen, which participants were able to see through a mirror positioned above their eyes.

**FIGURE 1:**
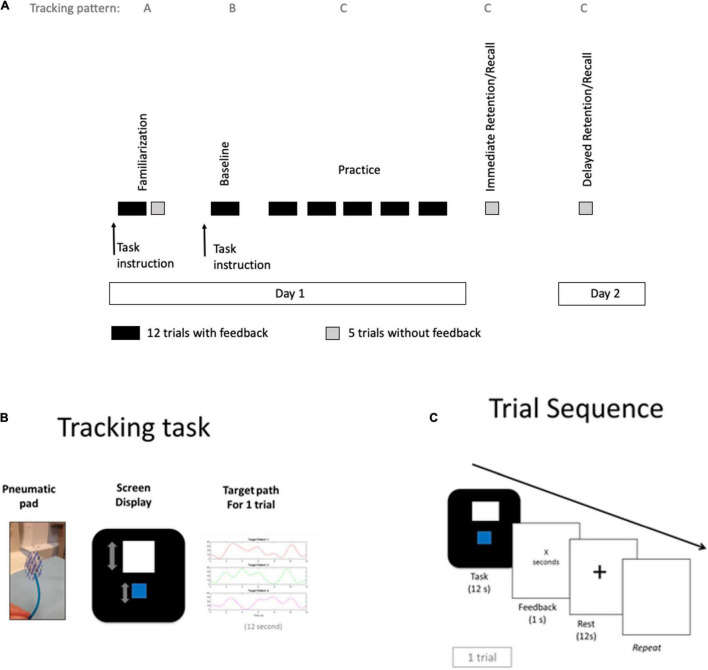
Study procedures, tracking task and trial sequence. **(A)** Study procedures started with Familiarization session in the mock scanner including trials with time on target feedback (dark rectangles) and test trials without feedback (gray squares), followed by a baseline block in the MRI scanner and 5 practice blocks. A test block was given in the scanner 5 min after end of practice (immediate retention). A second test block occurred 1 day later inside the mock scanner (Delayed retention). Three different spatiotemporal tracking patterns were used across time points (familiarization period: pattern A, baseline period: pattern B, and practice period: pattern C). **(B)** The tracking task involved applying force with thumb and index finger to a pneumatic pad that controlled the vertical movements of a blue cursor on the screen that displayed the moving white target, changing vertical position according to the 3 different patterns. **(C)** The sequence for each trial displayed 12 s of the task period, followed by a 1 s presentation of time on target feedback, which was followed by a 12 s rest period.

The study consisted of five phases (Familiarization, Baseline, Practice, Immediate Retention, and Delayed Retention), as illustrated in Figure 1. During the fMRI scan, all participants performed the tracking task over 6 blocks of 12 trials each (72 trials total), with KR performance feedback (time on target) presented after every trial. As illustrated in Figures 1B,C, all participants viewed a screen display with a cursor (blue box) and the target position (white box) which was updated every 20 ms during the 12 s task period; the degree to which the blue box was inside the white box throughout the target path reflected knowledge of performance feedback (KP) feedback. KP feedback was provided for all trials. The task period with KP was followed by a 1 s presentation of the total time on target (KR) in seconds over the 12-s trial. The KR was followed by a 12 s rest period and then the sequence (Task, Feedback, and Rest) repeated.

Prior to the fMRI session, participants were familiarized with the task inside a mock scanner (Familiarization), which was not part of the blocks performed in the real MRI scanner and not used in the imaging analysis (Figure 1A). The first block in the MRI scanner (Baseline) was used to assess baseline performance, followed by 5 practice blocks (Practice). Immediate retention performance was assessed with a test block (without KR feedback) 5 min after the practice session (Immediate retention) in the MRI and 1 day later (Delayed retention) inside the mock scanner. Both test blocks (immediate/delayed retention) included 5 trials without KR feedback. Participants did have KP feedback of the cursor and target positions during test blocks.

Three different spatiotemporal tracking patterns of equivalent difficulty were used to optimize comparability of tracking pattern and to dissociate non-pattern specific learning (i.e., learning generic control of the cursor with the pressure pad, but not specific to any one target pattern) for the three phases; Familiarization: pattern A, Baseline period: pattern B, and Practice period: pattern C (Figures 1A,B). All tracking patterns had equivalent amplitude range and number of reversals. For the exploratory aim, half of the participants received additional motivational statements verbally (see below for details) prior to each practice block (motivation plus feedback group) along with KR feedback after every trial. The motivational statements were designed to enhance intrinsic motivation during practice.

### Behavioral Task

The goal of the tracking task was to keep the cursor (blue box) inside a target box (white box), which was moving vertically across (up and down) the screen, for as long as possible in a 12-s trial period. Participants controlled the cursor movement by varying the isometric pinch force applied to an instrumented pneumatic pad (squeezing between the thumb and index finger). Performance information, specifically KR feedback, was presented for 1 s after each trial followed by a 12-s rest before the next trial began. All participants received the following instructions about the task: “In this task, there is a white target box that is moving in a specific pattern. The goal is to keep the blue box inside the white box for as long as possible. After every trial, you will see a number which is the amount of time the blue box was in the white box. Each trial is 12 s long. We will begin the trials now.”

### Behavioral Task Performance Measures

Task performance was assessed separately for acquisition and retention sessions in 2 ways. Time on target was used as one primary measure, which was part of the explicit task goal, and provided as information feedback (both KR and KP) during practice. For a second primary measure, we decomposed the participant’s cursor trajectory into spatial and temporal accuracy components using time-series analysis; the participant’s cursor trajectory was iteratively shifted in time toward the target onset until a maximum correlation between participant trajectory and target trajectory was achieved; this was done for each 12 s trial. The resulting correlation coefficient (after the temporal shift) represents the spatial accuracy of tracking performance. The number of milliseconds that the participant’s cursor trajectory was shifted to achieve the highest correlation coefficient (between the target and the cursor trajectory) represents the time lag or temporal accuracy of tracking.

#### Time on Target

The time that the cursor was entirely inside the target box was calculated for each trial. Average time on target was calculated separately for each trial block. If the participant kept the cursor in the target box for the entire length of the trial, a perfect score of 12 s would be displayed.

#### Spatial Accuracy: Maximum Correlation Coefficient

The maximum correlation coefficient between the target trajectory and the participant’s cursor trajectory, after adjusting for time lag, was calculated for each trial. Average correlation was calculated separately for each block. Perfect spatial accuracy would be reflected by *r* = 1.

#### Temporal Accuracy: Time Lag of Tracking

The shift in time that was associated with the maximum correlation between the target trajectory and participant’s cursor trajectory was calculated for each trial to determine temporal accuracy. Average time lag was calculated separately for each trial block. Perfect temporal accuracy reflected by anticipatory tracking would yield a time lag = 0. In practice, the shorter the time lag, the better the learner is anticipating the target pattern.

### Behavioral Data Analysis

To assess performance, time on target, maximum correlation of cursor and target trajectory (spatial accuracy) and the time lag that was used to achieve the maximum correlation (temporal accuracy) was evaluated for each block. Data were then corrected for outlier trials (time lag > average time lag of the corresponding block of trials ± 2 standard deviations).

### Motivational Statements

In addition to the basic task-related instructions, half the participants were given additional motivational statements (instructions and feedback) after the baseline block in the scanner. The motivation plus feedback group received the following oral motivational statements prior to each of the 5 practice blocks: prior to block (1) *Keep in mind that at the beginning it is common to undershoot or overshoot the target, but this is the type of task that you get better at with practice;* prior to block (*2) Alright! Your improvement across the past trials is reflecting your learning and getting the hang of it;* prior to block (3) *Very good! Based on your performance so far, it looks like you are going to continue to improve across the coming trials;* prior to block (4) *Alright! Nice work!*; prior to block (5) *Great job!* followed by *‘Well done’* at the end of the acquisition session. These motivational statements were modified from previous behavioral studies and were designed around three criteria: to enhance self-efficacy expectation, induce a positive (growth) concept of ability mindset and support perceived competence.

#### Self-Efficacy Questionnaire

A 6-item questionnaire assessed task-specific self-efficacy on a Likert scale. All participants were asked to rate how confident they were to keep the blue box (cursor) in the white target box for 2, 4, 6, 8, 10, and 12 s. The scale ranged from 0 (not confident at all) to 10 (extremely confident). The self-efficacy questionnaire was given after the familiarization block, before and after the practice phase, and before delayed retention trials on day 2. See Supplementary Material for questionnaire items.

#### Control Condition

Neutral statements (‘Just checking in, we will go ahead and continue with the task trials now.’) were provided to all participants between each of the 5 practice block runs to ensure that social contact and communication between the groups was comparable.

#### Intrinsic Motivation Inventory Questionnaire

An adapted version of the perceived competence, effort/importance and task interest/enjoyment subscale of the Intrinsic Motivation Inventory was administered after delayed retention trials on day 2. Participants rated their interest/enjoyment, perceived competence, and the effort/importance they attributed to the task on a Likert scale. Items on the questionnaire were shuffled in order of presentation for participants.

### Statistical Analysis of Behavioral Data

All statistical tests, with the exception of normal distribution tests, linear regressions, and linear mixed effects modeling, were conducted using SPSS. We checked for normal distribution using the Lilliefors test for normality. Time lag and self-efficacy data were normally distributed. Time on target and cross correlation were not normally distributed. For data that was not normally distributed (Time on target and cross correlation), we used linear mixed effects modeling. We used Matlab R2020b (MathWorks Inc., Natick, MA, United States) and its Statistical Toolbox for all regression analyses.

Levene’s test for equality of variance was used to check for verification of assumptions of homogeneity of variances. The time lag data with the exception of the practice 3 timepoint passed the test of homogeneity of variance. Time on target data for immediate and delayed retention tests did not show homogeneity of variance; thus, we used the Mann–Whitney *U* test.

To assess task improvement across practice for the time lag measure, performance during practice was analyzed in separate one-way repeated measures analysis of variance (rmANOVA) with practice block as the repeated measures factor. For the exploratory analysis (Aim 3), time lag performance during practice was analyzed in a 2 (groups: motivation plus feedback and feedback only) × 5 (practice blocks) ANOVA with repeated measures on the practice block factor, controlling for baseline performance.

To assess task improvement across practice and to compare task performance measures between the groups (Aim 3) for time on target and cross correlation, we used linear mixed effects modeling, with time points and groups as fixed effect variables and the baseline performance as a random effect variable. In this modeling, the time points and groups were set as categorical variables, and the baseline performance was set as a continuous variable. Specifically, we generated the linear mixed effects model using ‘fitlme’ function, then we used ‘anova’ function for the linear mixed effects model ANOVA marginal tests.

To compare groups on the time lag measure for immediate and delayed retention tests, two separate independent t-tests were used. To compare groups on the time on target and cross correlation measure, Mann–Whitney *U* test was used.

*Post hoc* tests were performed with a Bonferroni correction applied, resulting in a significance level set at *p* < 0.01 to adjust for multiple comparisons.

Self-efficacy was analyzed in a 2 (groups: motivation plus feedback and feedback only) × 4 (time point: baseline, pre practice, post practice and pre-delayed retention) repeated measures ANOVA to compare ratings between groups. We used a linear regression to examine the relationship between self-efficacy changes (before to after practice period) and time on target improvement (start to end of practice). We also used linear regression to test the linear relationship between self-efficacy changes (before to after practice period) and time on target, assessed at immediate and delayed retention, respectively.

An independent *t*-test was used to compare ratings between the groups for each subscale of the Intrinsic Motivation Inventory.

For all analyses, significance level was set at 0.05.

### Functional Magnetic Resonance Imaging Data Acquisition

We acquired T2-weighted echo spiral images with blood oxygen level dependent (BOLD) contrast using a 3 Tesla scanner (GE Signa Excite) with an 8-channel head coil. Each volume comprised 37, 3 mm thick axial slices, using the following parameters: TR = 2.5 s, TE = 34.5 flip angle = 90; FOV = 22, 64 × 64 matrix. For each participant, a total number of 131 volumes were obtained. Additionally, we acquired a T1-weighted structural image at the start of the scan session, before tracking task performance.

### Functional Magnetic Resonance Imaging Image Analysis

Analysis of fMRI data were conducted using FSL [FMRIB Software Library, (FSL, RRID:SCR_002823)]. The fMRI data for each participant were preprocessed using the FMRIB (Functional Magnetic Resonance Imaging of the Brain) Expert Analysis Tool (FEAT^1^). Preprocessing included skull extraction with the FSL brain extraction tool (BET), motion correction and spatial smoothing using a Gaussian kernel with full-width half-maximum of 5 mm.

For first level data analysis of each participant’s individual runs (block), we used a general linear model that included 2 regressors of interest for analysis: (1) onset and duration of each task period was defined for each trial (Task-related activity) (2) Performance-specific task activity included onset and duration of the task period that was defined for each trial, here specifically with parametric modulation of time on target performance for each corresponding trial (Performance-specific task activity) and a regressor of no interest: (3) performance feedback presentation period was modeled as a separate regressor with onset and duration corresponding to the 1 s feedback period following each task trial (Feedback-related activity).

To address Aim 1, the second regressor (Performance-specific task activity) was used to identify neural activity specifically modulated by time on target performance during acquisition. The first regressor (Task related activity) acted as a control to identify task period activity to enable comparison with other studies (e.g., Floyer-Lea and Matthews, 2004; Hatakenaka et al., 2007). For the higher-level analyses we combined the imaging data of the 5 practice blocks (i.e., 60 practice trials) and used a one sample *t*-test for each participant to assess Task-related activity and Performance-specific task-related activity across all participants.

To address Aim 2, the relationship between retention performance and acquisition phase brain activation was assessed using a single group average with an additional covariate, which was a behavioral measure of time on target performance at immediate retention. The group-level images were thresholded with cluster-based correction for multiple comparisons with *Z* > 2.3 and *p* < 0.05. Peak location for the local maxima within significant clusters are identified using MNI (Montreal Neurological Institute) atlas coordinates.

To address Aim 3, we used an independent samples *t*-test to compare brain activity between groups (motivation plus feedback group and feedback only group) (1) related to the task and (2) related to performance-specific task-activity (contrast 1: associated with higher time on target; contrast 2: associated with lower time on target).

Six participants were excluded from the fMRI analysis (Motivation plus feedback group: 1 excluded due to excessive head motion, 1 incidental finding of a congenital neurological condition and Feedback only group: 3 excluded due to excessive head motion; 1 missing neuroimaging data), leaving *n* = 18 in the motivation plus feedback group and *n* = 16 in the Feedback only group represented in the reported fMRI results. Data from all 40 participants were used for the behavioral measures analysis.

## Results

### Behavioral Measures of Motor Performance and Learning

#### Task Performance Improved With Practice and Was Retained

**FIGURE 2:**
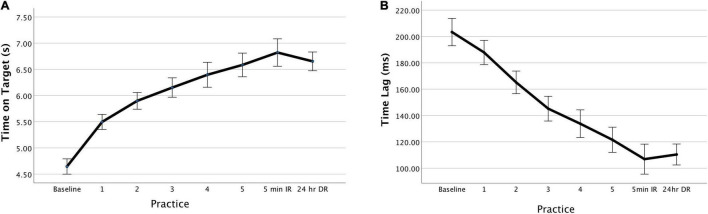
Task performance improves with practice. Group average showing performance curve for **(A)** time on target and **(B)** temporal accuracy (time lag). IR, immediate retention; DR, delayed retention. Error bars = ±1 standard error (*N* = 40).

Participants improved performance across practice blocks as evidenced by time on target, *F*(1,187) = 18.28, *p* < 0.001 (Figure 2A) and time lag (temporal accuracy), *F*(1,36) = 48.93, *p* < 0.001 (Figure 2B), but not for cross correlation (spatial accuracy), *F*(4,188) = 0.632, *p* = 0.64 (Supplementary Figure 5A). Furthermore, as illustrated in Figure 2 and Supplementary Figure 5A, performance at immediate retention was maintained to a level comparable, if not slightly better than that achieved at the end of the practice period.

### Brain Activation Associated With Motor Performance and Learning

#### Task-Related Brain Activity During Practice

**FIGURE 3:**
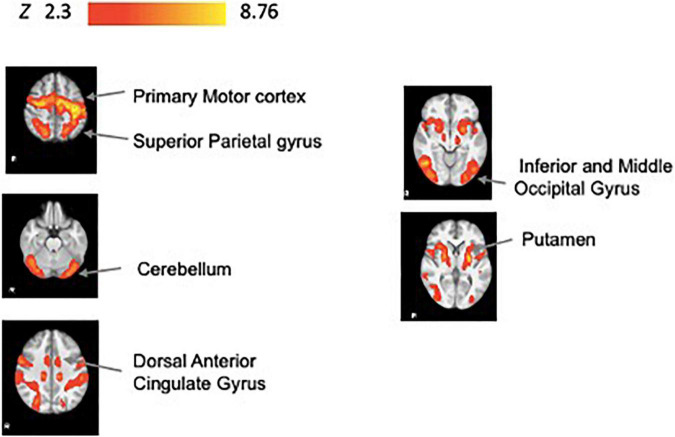
Task-related fMRI activity. Brain activity associated with task period during practice (*N* = 34). We show significant associations in the precentral gyrus, dorsal anterior cingulate gyrus, superior parietal gyrus, putamen, fusiform gyrus, inferior and middle occipital gyrus, and superior parietal gyrus. All results have been corrected for multiple comparisons (*p* < 0.05).

Neural correlates of motor execution during the task period (Task-related activity) were principally observed in the motor cortex, dorsal anterior cingulate gyrus, parietal gyrus, putamen, occipital gyrus, and the cerebellum (Figure 3 and Supplementary Figure 2 for slice-by-slice whole brain activation maps). All reported significant activations (Table 2) have a minimum of 100 voxels, with a threshold of *p* < 0.05.

**TABLE 2:** Summary of primary fMRI activation clusters during practice (task-related activity).

Cluster size (# voxels)	Cluster *p*-value	Peak		Anatomical location (side)
		*Z-score*	*x-, y-, z- (mm)*	
31393	1.93e-33	8.76	−38, −18, 50	Precentral gyrus (L)
		7.76	−24, −12, 54	
		7.6	−24, −16, 48	
		7.9	−8, −9, 46	Dorsal anterior cingulate gyrus (L)
		7.73	−38, −36, 46	Superior parietal gyrus (L)
		7.54	−26, −6, 0	Putamen (L)
11232	2.11e-16	7.42	48, −64, −14	Fusiform gyrus (R)
		7.28	48, −62, −10	Inferior occipital gyrus (R)
		6.5	26, −70, 36	Middle occipital gyrus (R)
		6.42	22, −68, 52	Superior parietal gyrus (R)
		6.28	26, −66, 56	
		6.04	38, −78, −14	Inferior occipital gyrus

*The listed clusters showed significant activation (p < 0.05) across task trials. Local maxima for each cluster have been identified separately. Effect sizes of BOLD signal increase during task period are reported. Effect size expressed as mean beta values. Z values indicate the local maxima at the ROI center. L, left; R, right. Peak location for the local maxima are MNI atlas coordinates.*

#### Performance-Specific Task Related Brain Activity During Practice

**FIGURE 4:**
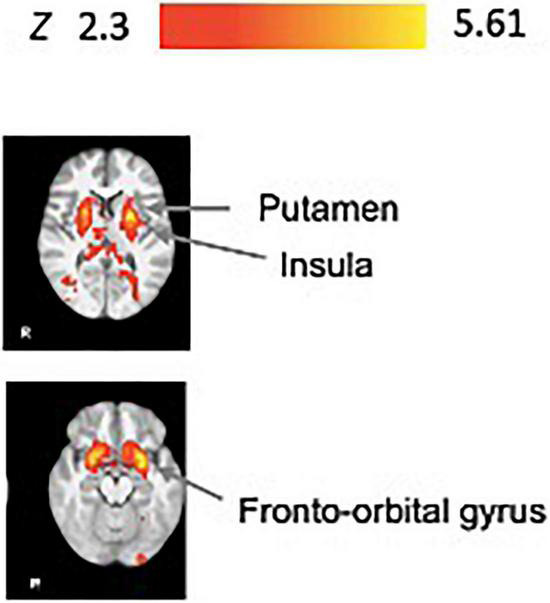
Performance-specific task fMRI activity. Brain activity associated with performance improvements during practice (*N* = 34). We show significant associations in the putamen, fronto-orbital gyrus, amygdala, insula, and uncinate fasciculus. All results have been corrected for multiple comparisons (*p* < 0.05).

Figure 4 displays the Performance-specific task activity. As illustrated, trials with relatively long time on target performance (i.e., higher accuracy) were significantly associated with activation in the frontal orbital cortex, putamen, amygdala, and insula (Figure 4, Table 3 and Supplementary Figure 3 for slice by slice whole brain activation maps).

**TABLE 3:** Summary of primary fMRI activation clusters associated with performance during the practice blocks (Performance-specific task activity).

Cluster size (# voxels)	Cluster *p*-value	Peak		Anatomical location (side)
		*Z-score*	*x-, y-, z- (mm)*	
22741	1.69e-32	5.61	−24, 2, 6	Putamen (L)
		5.5	22, 6, 0	Putamen (R)
		5.34	22, 10, −22	Fronto-orbital gyrus (R)
		5.29	−26, 4, −16	Amygdala (L)
		5.21	−26, 10, −16	Insula (L)
		5.18	−28, 2, −12	Uncinate fasciculus (L)

*The listed clusters showed significant activation (p < 0.05) across task trials. Local maxima for each cluster have been identified separately. Effect sizes of BOLD signal increase during task period are reported. Effect size expressed as mean beta values. Z-values indicate the local maxima at the ROI center. L, left; R, right. Peak location for the local maxima are MNI atlas coordinates.*

#### Brain Regions Active During Practice That Are Associated With Immediate Retention Performance

**FIGURE 5:**
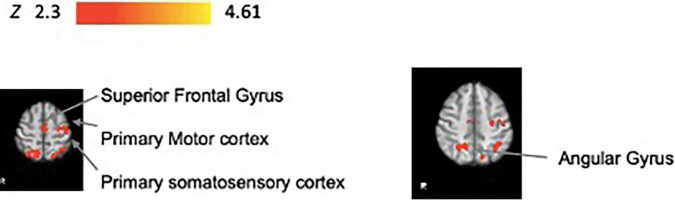
Brain activity during task practice that is correlated with immediate retention performance (*N* = 34). We show significant associations in the postcentral gyrus, superior frontal gyrus, precentral gyrus, superior parietal gyrus, and angular gyrus. All results have been corrected for multiple comparisons (*p* < 0.05).

Figure 5 illustrates the significant areas of activity during practice that are associated with performance during the no-KR immediate retention test. These areas included: primary motor cortex, superior frontal gyrus, somatosensory cortex, angular gyrus, and parietal gyrus (Table 4 and Supplementary Figure 4 for slice by slice whole brain activation).

**TABLE 4:** Summary of primary fMRI activation clusters that are associated with immediate retention performance.

Cluster size (# voxels)	Cluster *p*-value	Peak		Anatomical location (side)
		*Z-score*	*x-, y-, z- (mm)*	
1782	5.69e-05	4.61	−46, −16, 58	Postcentral gyrus (L)
		3.46	−40, −24, 62	
		3.5	−40, −14, 68	
		4.57	−4, −12, 66	Superior frontal gyrus (L)
		3.94	−28, −14, 44	Precentral gyrus (L)
		3.84	−8, −14, 70	
906	0.00433	4.87	14, −52, 52	Superior parietal gyrus (R)
		4.5	16, −64, 60	
		4.28	28, −60, 54	
		3.92	32, −66, 58	
		3.8	14, −66, 66	
686	0.0156	4.12	−18, −68, 68	Superior parietal gyrus (L)
		3.74	−22, −68, 64	
		3.77	−40, −50, 42	Angular gyrus (L)
		3.55	−30, −70, 62	
		3.49	−36, −56, 52	

*The listed clusters showed significant activation (p < 0.05) across task trials. Local maxima for each cluster have been identified separately. Effect sizes of BOLD signal increase during task period are reported. Effect size expressed as mean beta values. Z-values indicate the local maxima at the ROI center. L, left; R, right. Peak location for the local maxima are MNI atlas coordinates.*

### Impact of Motivational Statements on Task Performance

#### Motivational Statements Improve Task Performance During Practice

**FIGURE 6:**
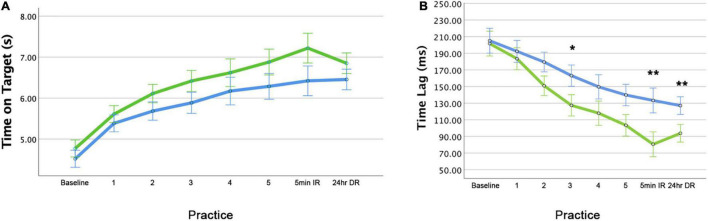
Motivational statements improve task performance. Motivational statements improve **(A)** time on target and **(B)** temporal accuracy (time lag) performance during practice and retention (motivation plus feedback = green, feedback only = blue). *p < 0.05, **p < 0.01 for between group comparisons. Note that the significance level was corrected for multiple comparisons for practice blocks. IR, immediate retention; DR, delayed retention. Error bars = ± 1 standard error.

Baseline performance was similar in both groups for time on target (Figure 6A), temporal accuracy (time lag) (Figure 6B) and spatial accuracy (cross correlation) (Supplementary Figure 5B). There was no statistically reliable effect of group during practice for time on target *F*(1,187) = 0.21, *p* = 0.27 or spatial accuracy, *F*(1,188) = 0.99, *p* = 0.32 (Figure 6 and Supplementary Figure 5B). Also, there was no Group × Block interaction for time on target, *F*(4,187) = 0.65, *p* = 0.62 or spatial accuracy performance, *F* (4,188) = 1.20, *p* = 0.31.

Notably, there was a reduction in time lag of tracking for both groups, with the motivation plus feedback group showing greater temporal accuracy during acquisition (Figure 6B). This group difference was reliable as revealed by a statistically significant group main effect, *F*(1,34) = 5.02, *p* = 0.03. There was no Group x Block interaction for this aspect of performance, *F*(1,34) = 1.74, *p* = 0.20.

#### Motivational Statements Improve Temporal Accuracy at Immediate and Delayed Retention

The motivation plus feedback group had significantly lower time lag (higher temporal accuracy) at immediate retention, *t*(37.82) = −2.96, *p* = 0.01, and delayed retention, *t*(29.74) = −2.80, *p* = 0.01, compared with the feedback only group. (Figure 6B). Figure 6A shows that compared with the feedback only group, the motivation plus feedback group demonstrated higher time on target performance at immediate and delayed retention, *U* = 140, *p* = 0.11 and *U* = 131, *p* = 0.22, respectively; though, this was not statistically significant. The groups show comparable spatial accuracy for immediate and delayed retention, *U* = 165, *p* = 0.48 and *U* = 188, *p* = 0.61, respectively (Supplementary Figure 5B).

#### Self-Efficacy Change Over Practice Significantly Explained the Variance in Retention Performance in the Motivation Plus Feedback Group, but Not the Feedback Only Group

Participants increased self-efficacy rating across time points, *F*(1,34) = 44.06, *p* < 0.001. The motivation plus feedback group showed a trend for higher self-efficacy ratings post practice and pre-delayed retention compared to the feedback only group; yet this difference was not statistically significant, *F*(1,33) = 1.80, *p* = 0.19 (Supplementary Figure 1B). In addition, interest/enjoyment and perceived competence ratings on the Intrinsic Motivation Inventory were comparable between groups, *t*(35) = 0.96, *p* = 0.33 and *t*(35) = 0.95, *p* = 0.35, respectively (Supplementary Figure 6). Effort/importance ratings were marginally higher in the motivation plus feedback group, approaching statistical significance *t*(35) = 1.79, *p* = 0.08.

Across the groups, there was a significant linear relationship between change in self-efficacy (pre to post training) and gain in time on target performance during practice (gain = difference between baseline and last training block), *R*^2^ = 0.301, *F*(1,34) = 14.61, *p* = 0.002 and this relationship was similar between motivation plus feedback and feedback only groups [motivation plus feedback: *R*^2^ = 0.40, *F*(1,15) = 9.98, *p* = 0.007, feedback only: *R*^2^ = 0.27, *F*(1,17) = 6.12, *p* = 0.02], (not shown).

**FIGURE 7:**
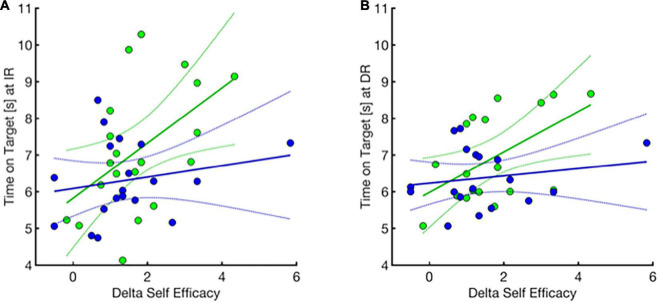
Relationship of delta self-efficacy change (*x*-axis) and motor performance (*y*-axis) at immediate and delayed retention for motivation plus feedback (green) and feedback only (blue) groups. Pre to post practice change in self-efficacy is correlated with higher retention performance at immediate and delayed retention in the group that receives motivational statements in addition to performance feedback compared to feedback alone. **(A)** Immediate retention performance. Two participants had missing data and were excluded from data analysis. (1 missing pre-training time point, and 1 missing post-training time point, were removed from correlation analysis). **(B)** Delayed retention performance. Three participants had missing data and were excluded from data analysis. (3 missing delayed retention time point, were removed from data analysis). Dotted lines indicate the 95% confidence interval.

Importantly and illustrated in Figure 7, there was a statistically significant linear relationship between pre-to-post practice change in self-efficacy (Delta self-efficacy) and time-on-target performance at immediate (Figure 7A) and delayed retention (Figure 7B), for the motivation plus feedback group (green), but not for the feedback only group (blue), [immediate retention, motivation plus feedback: *R*^2^ = 0.25, *F*(1,18) = 6.07, *p* = 0.02, feedback only: *R*^2^ = 0.04, *F*(1,17) = 0.75, *p* = 0.40; Delayed retention, motivation plus feedback: *R*^2^ = 0.28, *F*(1,16) = 6.24, *p* = 0.02, feedback only: *R*^2^ = 0.03, *F*(1,16) = 0.56, *p* = 0.47].

#### Motivational Statements Effect on Functional Magnetic Resonance Imaging Brain Activity Was Not Observed

We observed no group differences in brain activity during the task practice period. Moreover, there were no brain activity differences related to Performance-specific task activity nor brain activity during task practice that were related to immediate retention performance.

## Discussion

While recent contemporary frameworks have identified neural regions involved with specific mechanisms of sensorimotor encoding and recall, this is still a new frontier in behavioral neuroscience seeking a better understanding of the “neural implementation of behavior” that allows for motor skill learning (Krakauer et al., 2017). We designed a study that captured brain activity through whole brain fMRI during acquisition/practice (encoding) and utilized individual behavioral performance metrics (i.e., time on target) data to investigate the neural learning mechanisms that implement performance improvements during practice and performance at immediate retention.

In particular, we examined how task performance modulates brain activation during motor skill acquisition (distinct from the more common approach that captures general task planning and execution processes i.e., task-related activity). Additionally, we aimed to examine the neural implementation during the dynamic process of encoding and memory formation that are reflected through retention performance (recall). Finally, we explored the influence of carefully crafted motivational statements during motor skill acquisition on brain and behavior mechanisms of motor learning.

This study adds to the literature by demonstrating that the neural implementation of practice improvement during encoding is associated with primarily cortical and subcortical brain regions, while activity in cortical regions is specifically correlated with immediate retention performance. The unique contribution of our approach allowed us to isolate the neural activity patterns associated with the performance-specific analysis from the typical task-specific analysis thereby revealing the distinct nature of performance modulated neural activity that allows for motor learning. We highlight this revelation in the next two sections.

### Task-Related Activation Is Consistent With Visuomotor Task Practice Demands

The neural activation we identified through our Task-related practice activity analysis aligns with the four learning mechanisms we expect with our visuomotor tracking task paradigm: cognitive strategy learning (prefrontal), reinforcement learning (basal ganglia), use dependent learning (motor cortex) and error-based learning (cerebellum) mechanisms. Moreover, these findings are consistent with previous studies that have examined the learning of implicit motor skills such as visuomotor tracking and isometric force production (Honda et al., 1998; Grafton et al., 2002; Vaillancourt et al., 2003; Floyer-Lea and Matthews, 2004, 2005; Hatakenaka et al., 2007; Dayan and Cohen, 2011).

### Performance-Specific Task Activity Associated With Corticostriatal Activation Required for Skill Acquisition

Our findings for the activation correlated specifically with performance during task practice supported our hypothesis for engagement of the prefrontal cortex and striatal regions (Figure 4). Considering the motor planning that is inherent in the feedforward processes of learning the tracking pattern, these processes likely engage the cognitive strategy learning mechanism to allow effective tracking of the target, reflected in higher time on target performance. Specifically, the cognitive strategy engaged to follow the task instructions (“to keep the cursor within the moving target box for as long as possible”) and develop plans to achieve the task goal requires frontal cortical regions as one applies explicit knowledge of the task goal. This idea is consistent with our results showing frontal orbital cortex activity associated with motor task improvement during practice. The prefrontal cortex, including the frontal orbital cortex, has previously been implicated in motor planning of goal directed movements and suggested in implementing cognitive strategy processes in early motor learning (O’Doherty, 2011; Spampinato and Celnik, 2021).

Furthermore, our task paradigm involves the provision of performance feedback (KR) after each trial in addition to visual feedback of the cursor and target position (KP), both engaging reinforcement learning processes. Reinforcement learning is defined by the process of selecting appropriate motor actions based on feedback about the success or failure of a movement to maximize reward (i.e., score, KR performance feedback) (Sutton and Barto, 2018). Animal and human studies have identified the basal ganglia and dopaminergic system as providing a critical role in action selection and reinforcement learning (Schultz, 2016). Specifically, human neuroimaging studies report that the corticostriatal circuit, including activation in the ventral tegmental area, the striatum and prefrontal cortex (including the frontal orbital cortex), are critical for motor learning, and these areas are experimentally activated by performance feedback and monetary reward (Dayan and Balleine, 2002; Wickens et al., 2003; Pessiglione et al., 2007; Wrase et al., 2007; Schmidt et al., 2009; Wächter et al., 2009; Lutz et al., 2012; Widmer et al., 2016; Becker et al., 2017). Animal work further supports the contribution of the basal ganglia to motor cortex plasticity in reinforcement learning of motor skills (Molina-Luna et al., 2009; Hosp et al., 2011; Hosp and Luft, 2013). Further, activity in the basal ganglia, and the putamen are associated with performance of learned movement sequences—this aligns well with our task demands.

### Differences Between Task-Related Activity and Performance Specific Task Activity

The brain region associated with error-based learning, also termed sensorimotor adaptation, notably the cerebellum, is not represented in our analysis of Performance specific task activity. One reason for this may be that there is ample practice (24 trials during familiarization and the baseline periods combined) with 2 other tracking patterns before the pattern-specific practice period begins. For the Performance specific task analysis, we specifically examined brain activity associated with trial-by-trial task performance during the acquisition phase. We suspect that much of the non-specific learning of how to perform the task, which engages error-based processes, such as calibration of one’s force with the grip of the pad to control the cursor, may have occurred in those early familiarization trials prior to the task acquisition phase. This finding is different from our analysis of task-related activity (without performance modulation), where we do see cerebellar activity. Thus, one viable interpretation is that cerebellar activity is relevant to execution of the task throughout practice as seen in our task related activity analysis, reflecting non-specific learning processes (i.e., adaptation) for these kinds of continuous tracking tasks.

### Cortical Activity Promotes Motor Retention Performance

Our findings showing cortical activity, including the primary motor cortex, supports our hypothesis for the role of the motor cortex in predicting immediate retention performance. The regions engaged, including primary motor cortex, superior frontal gyrus, somatosensory cortex and angular and parietal gyrus are consistent with work that has begun to investigate neural activity associated with next day motor retention. Recent studies in humans that use non-invasive brain stimulation reveal that M1 excitability during practice is proportional to retention performance the next day (Cantarero et al., 2013; Spampinato and Celnik, 2017; Uehara et al., 2018). As noted in the Introduction, a human study that combined TMS and fMRI imaging found increased prefrontal, premotor and parietal areas in addition to increased M1 excitability during practice that together, correlated with retention of a serial reaction time sequence learning task (Lin et al., 2011).

Together, these studies emphasize the relevance of cortical region activity during acquisition that is correlated with retention performance. These cortical regions are all relevant for motor planning, preparation, and controlled sequencing of movements important for learning the track pattern and reflected by retention performance. Such learning processes are critical in acquiring the skills during practice that lead to and can predict successful recall performance during retention tests. Specifically, the feedforward nature of learning to anticipate the target trajectory that allows one to maximize the time on target during each trial (quantified by a reduction in time lag across practice) is consistent with prefrontal and motor cortical activity that we found to be critical to the prediction of immediate retention performance.

Furthermore, neuroimaging studies demonstrate that the angular gyrus has an important role in perceptual learning and spatial action awareness, specifically in identifying discrepancies between an intended action and movement consequences (Grafton et al., 2002). There is evidence that the angular gyrus is engaged with attention control and visuospatial navigation. For example, a human study revealed the role of the angular gyrus in shifting attention to salient stimuli and mediating the allocation of attention to task-relevant information (Gottlieb, 2007). These previous studies provide evidence that the angular gyrus activity may have a role in directing the attention required to learn a 12-s target pattern and thereby support improved anticipatory feedforward processes that reduce time lag, increase time-on-target (success), and predict more effective immediate retention performance (Seghier, 2013). It is also possible that engagement of this region is relevant to performing the task effectively including keeping track of the position of the cursor relative to the target in space and to maintain the cursor position within the target during the task trials. Effectiveness of these processes during practice may be reflected in a relatively high time-on-target performance at immediate retention when performance feedback is withheld and thus visual feedback of the cursor and target position during retention trials becomes more salient.

We speculate that a greater understanding of the brain regions active during encoding, that predict greater recall performance, may show promise toward the development of a diagnostic biomarker that can be used for individuals approaching neurorehabilitation. An assessment of the degree of existing neural substrates engaged during task acquisition, that are important for effective motor learning (i.e., recall), may inform the potential success of a given treatment plan.

Finally, the absence of basal ganglia activity in the brain regions found to be associated with immediate retention performance is supported by research that highlights the significant and necessary contribution of the dopaminergic system to the motor cortex specifically for skill acquisition during the encoding phase. Animal studies have revealed that while dopaminergic projections to the motor cortex are necessary for skill acquisition, these connections are not necessary for recall performance after the acquisition period is complete (Molina-Luna et al., 2009; Hosp et al., 2011; Hosp and Luft, 2013).

These results underscore the importance of cortical activation during skill acquisition of visuomotor tasks for long term retention of motor skills and its potential for interventions that may capitalize on these regions to maximize motor skill learning and immediate retention. Our Performance specific activation analysis corroborates the crucial role of basal ganglia for encoding processes and its unique role in performance improvements during acquisition. This emphasizes the nature of motor learning processes such as reinforcement learning (modulated by the basal ganglia) in promoting cognitive strategies and use-dependent practice mechanisms, dependent on key neural nodes required for learning the task-specific skills (namely the prefrontal and motor cortices). Translating these findings suggests that primary attention to conditions during learning, including strategy based, explicit instructive feedback and practice schedules (e.g., contextual interference) that support engagement of the prefrontal and motor cortices during practice can foster greater retention of motor skills, while reinforcement-based interventions, such as motivational statements and feedback, can boost and support those principal motor learning processes.

This understanding may be crucial for the development of interventions to promote motor recovery in populations with neurological disorders. Given that recovery-supportive neuroplasticity, motor learning and task practice are fundamental to neurorehabilitation (Winstein et al., 2014) understanding how to engage the neural substrates associated with contemporary motor learning mechanisms will serve to begin to translate this new science into actionable practice through evidence-based clinical decision making (e.g., Leech et al., 2022). Our study findings provide beginning evidence to inform the design of interventions that incorporate task practice paradigms in light of the neural substrates engaged in motor learning. For example, a patient with cerebellar damage may benefit from therapeutic treatments that target prefrontal and motor cortical areas, through strategy based and use-dependent learning processes, rather than those that are heavily dependent on error-based or adaptation learning processes. Future research is needed to test and eventually realize these kinds of speculations and to build the evidence base that can inform clinical decision making by effectively integrating these motor learning mechanisms for individualized cases with varying functional, neurological and psychological needs.

### Motivational Statements Promote Motor Performance and Learning but This Benefit Is Not Observed in the Functional Magnetic Resonance Imaging Brain Activity Signal

Our behavioral findings from the between group (motivation plus feedback vs. feedback alone) analyses show that statements to enhance intrinsic motivation and supplement performance feedback improve motor performance and learning of a visuomotor tracking task. This is evidenced by improved temporal accuracy and time-on-target measures (Figure 6). The significant benefit was evidenced by shorter tracking time lag (higher temporal accuracy) both during acquisition and through immediate and delayed retention performance.

The actual time lag during immediate and delayed retention was ∼90 ms for the motivation plus feedback group compared to ∼130 ms for the feedback only group, which is nearly 40 ms shorter. This is a clear indication that the motivational statements enlisted enhanced anticipatory processes, and importantly supported better memory development for the tracking pattern than achieved with KR/KP feedback alone. The skill to anticipate the upcoming position of the target and then to coordinate grip pressure accordingly demonstrates an elegant motor planning and execution capability.

Our behavioral findings are consistent with previous studies reporting greater automatic movement control provided through instructions that enhanced learners’ sense of competence (Chiviacowsky et al., 2012). Previous behavioral studies provide evidence for the benefits to motor skill learning and performance of various manipulations designed to induce both intrinsic and extrinsic forms of motivation. These manipulations include monetary reward (Abe et al., 2011; Galea et al., 2015), social comparison (Lewthwaite and Wulf, 2010), social reward (Sugawara et al., 2012), performance feedback (Chiviacowsky and Wulf, 2007) and instructions that enhance expectancies and support competence (Wulf and Lewthwaite, 2009; Chiviacowsky et al., 2012; Wulf et al., 2014). In a 2016 theoretical review paper, Wulf and Lewthwaite propose a new motor learning theory, OPTIMAL (Optimizing Performance through Intrinsic Motivation and Attention for Learning), in which intrinsic motivation critically contributes to skill learning by strengthening the task goal action coupling (Wulf and Lewthwaite, 2016). Taken together, enhancing intrinsic motivation as implemented here may have improved implicit skill learning in part through the promotion of a focus on the task goal, a reduction of attention to self-regulatory processes (that may interfere with learning) and an optimization of the link between the task goal and the sequence of actions necessary to achieve that goal. Moreover, aspects of intrinsic motivation including effort/importance attributed to the task can also be influenced by the motivational statements as supported by the trend for higher effort/importance ratings reported by the motivation plus feedback group (Supplementary Figure 6). This effect may be critical for motor skills that require dynamic and delicate motor control such as playing a musical instrument, operating a medical tool, or recovering compromised motor control through neurorehabilitation, where learning can be improved through incorporation of motivational statements during training (Gangwani et al., 2022).

Our self-efficacy findings provide further evidence that the motivational statements did indeed have the intended impact on performance and especially retention performance. The significant linear relationship between self-efficacy enhancement and immediate retention performance for the motivation plus feedback group, but not the feedback only group, may suggest the nature of the impact of KR/KP feedback on retention performance when motivational statements are provided. While both groups received performance feedback, the motivation plus feedback group may apply more salience to the feedback, making it a stronger reinforcement compared to feedback alone and thereby influence encoding processes which are also reflected by performance gains at immediate and delayed retention (i.e., enhanced temporal accuracy). We speculate that this could be likened to the beneficial learning effects seen when an extrinsic motivator (i.e., monetary reward) is coupled with performance feedback (Widmer et al., 2016; Sporn et al., 2021; Vassiliadis et al., 2021). Exploration of other factors of intrinsic motivation, including pressure/tension and perceived choice, may provide a fuller view of how motivational statements influence motor behavior. Furthermore, there is related research to support the value of providing enhanced feedback during practice to promote motor recovery (Subramanian et al., 2013; Popović et al., 2014). For instance, a recent study investigating the effects of arm retraining using virtual reality after stroke reported advantages of using a virtual environment as compared to a physical environment on arm motor recovery. These findings suggest that feedback is more effectively used when enhanced through a virtual environment, possibly due to heightened salience of the feedback provided and greater awareness of the task success during practice (Subramanian et al., 2013). Such work aligns with our findings here and provides preliminary evidence for the benefits of a set of motivational interventions including virtual reality as a supplement to traditional information feedback to promote motor learning during the course of neurorehabilitation.

The lack of brain activation correlates for the robust behavioral findings was surprising. However, in retrospect, there are several reasons for why we may not have seen group differences in the fMRI signal between those who received motivational statements plus performance feedback and those who received feedback alone. Firstly, both groups show task performance improvements and self-efficacy rating increases throughout practice and retention test time points. We controlled many of the variables amongst the participants to examine neural correlates of practice and retention performance across all individuals, including a common task, comparable informational performance feedback, consistent practice structure, and the same number of trials. Most relevant to interpretation of the neural effects of motivational statements is that all participants received KR and KP (visual feedback of the cursor and target position) which as we have discussed earlier can also act as a source of reinforcement and motivation (success of movement) and thus may have engaged neural circuitry that overlaps and thus limits the sensitivity to pick up additional motivation-related engagement of those same neural circuits through our fMRI study design. Moreover, the results from the direct measure of intrinsic motivation (i.e., IMI) suggest that the differences in intrinsic motivation may not have been robust enough to see between-group differences in fMRI activation. Using the three subscales of the intrinsic motivation inventory (interest and enjoyment, perceived competence and effort/importance), we observed a trend for greater effort/importance ratings reported in the motivation plus feedback group compared to the feedback only group. However, we did not see statistically significant between-group differences in IMI ratings for any of the three subscales. Results from our first level fMRI analysis that identified the neural correlates of performance improvements supports the possibility of performance feedback acting on motivational processes that are inherent to reinforcement learning. Specifically, the striatal regions involved with encoding task components are correlated with better practice performance. Such brain activation findings are aligned with human studies that have investigated the role of motivational circuits in motor skill learning (Pessiglione et al., 2007; Wrase et al., 2007; Schmidt et al., 2009, 2012; Wächter et al., 2009; Lutz et al., 2012; Widmer et al., 2016) and recently, the initiation of movement (Hamilos et al., 2021). The comparable paradigm factors and study conditions may have reduced the between-group differences in fMRI neural correlates mediating the boost in behavior we did observe for the motivation plus feedback group.

Further, it is possible that other motor tasks and populations (e.g., full body balance tasks; older individuals) different task demands, and/or experimental paradigms may enable a more robust neural activation response to instructions of enhanced intrinsic motivation (Nandi et al., 2019). For example, a within-subject design where statements (i.e., motivational, neutral) are provided in a pseudo-random order may have been more appropriate for our aim. Further, it is also possible that a differential neural effect may have been picked up at the retention phases when the behavioral group differences were more robust. Future studies should explore the brain correlates at all testing periods (immediate and delayed retention time points) to examine if brain activation associated with retention performance may be more sensitive to the effects of intrinsic motivation enhancements applied during acquisition.

Finally, we utilized an fMRI block design, which comes with the disadvantage of potential habituation, anticipation, and other strategy effects across the scanning period. While we tried to address the possibility for habituation and anticipation during the scanning period to influence the between-group comparisons by having a feedback only group, further studies could better address this limitation using event-related neuroimaging paradigms. Additionally, other neuroscientific methods such as EEG, PET, or TMS or a combination (e.g., Lin et al., 2011) may be more sensitive to the multi-faceted effects of motivational statements on neural networks that drive the behavioral enhancements we observed here (Meadows et al., 2016).

## Conclusion

Our findings provide evidence that performance during skill acquisition (i.e., encoding) and retention (i.e., recall) are associated with distinct neural components of the four major motor learning mechanisms engaged with practice of a visuospatial pinch force tracking task. While cortical activity, including motor and prefrontal cortices (cognitive strategies) was correlated with retention performance, both cortical and subcortical area activity, namely prefrontal (cognitive strategies) and striatal regions (reinforcement learning), were associated with task performance improvement during practice. Results from our exploratory analysis of the influence of intrinsic motivational enhancements on motor learning mechanisms demonstrate enhanced anticipatory performance implemented through more precise timing of movements during acquisition and retention performance. These behavioral findings are comparable to studies utilizing extrinsic motivational manipulations (i.e., monetary reward) during motor learning.

## Data Availability Statement

The raw data supporting the conclusions of this article will be made available by the authors, without undue reservation, to any qualified researcher.

## Ethics Statement

The studies involving human participants were reviewed and approved by University of Southern California (USC) Institutional Review Board. The patients/participants provided their written informed consent to participate in this study.

## Author Contributions

DB-K: conception and design of the study, fMRI data analysis, statistical data analysis, writing—original draft preparation, and data visualization. BK: fMRI data analysis, statistical data analysis, data visualization, and editing. JM: conceptualization, supervision, fMRI analysis, and writing—review and editing. RL and CW: conceptualization, supervision, and writing—review and editing. All authors contributed to manuscript revision, read, and approved the submitted version.

## Conflict of Interest

The authors declare that the research was conducted in the absence of any commercial or financial relationships that could be construed as a potential conflict of interest.

## Publisher’s Note

All claims expressed in this article are solely those of the authors and do not necessarily represent those of their affiliated organizations, or those of the publisher, the editors and the reviewers. Any product that may be evaluated in this article, or claim that may be made by its manufacturer, is not guaranteed or endorsed by the publisher.

## Footnotes

1
http://fsl.fmrib.ox.ac.uk/fsl/fslwiki/

## Supplementary Material

Supplementary Figure 1 Self-Efficacy rating across time points for **(A)** all participants (*N* = 40) and **(B)** motivation plus feedback (green) and feedback only (blue) groups.

Supplementary Figure 2 Slice-by-slice whole brain activation from Task-related activity analysis.

Supplementary Figure 3 Slice-by-slice whole brain activation from Performance specific task activity analysis.

Supplementary Figure 4 Slice-by-slice whole brain activation during practice that is associated with immediate retention performance.

Supplementary Figure 5 Spatial accuracy (maximum cross correlation) during practice and retention for **(A)** all participants (*N* = 40) and **(B)** between groups (motivation plus feedback = green, feedback only = blue). IR, immediate retention; DR, delayed retention. Error bars = ± 1 standard error.

Supplementary Figure 6 Intrinsic motivation inventory subscale rating for motivation plus feedback (green) and feedback only (blue).
