# Supplementary material for: Different Patterns of Neural Activity Characterize Motor Skill Performance During Acquisition and Retention
Source: Front Hum Neurosci. 2022 Jun 13;16:900405. doi: 10.3389/fnhum.2022.900405 (PMC9234574; doi:10.3389/fnhum.2022.900405)
Supplement: Supplementary file 1 [file Table_1.DOCX]

**Research Study Questionnaire**

*Please indicate how much you agree with the statement.*

Please rate how confident you are that you would be able to keep the red box in the white target box for 2 seconds?

0 1 2 3 4 5 6 7 8 9 10

(Not confident at all) (Extremely confident)

Please rate how confident you are that you would be able to keep the red box in the white target box for 4 seconds?

0 1 2 3 4 5 6 7 8 9 10

(Not confident at all) (Extremely confident)

Please rate how confident you are that you would be able to keep the red box in the white target box for 6 seconds?

0 1 2 3 4 5 6 7 8 9 10

(Not confident at all) (Extremely confident)

Please rate how confident you are that you would be able to keep the red box in the white target box for 8 seconds?

0 1 2 3 4 5 6 7 8 9 10

(Not confident at all) (Extremely confident)

Please rate how confident you are that you would be able to keep the red box in the white target box for 10 seconds?

0 1 2 3 4 5 6 7 8 9 10

(Not confident at all) (Extremely confident)

Please rate how confident you are that you would be able to keep the red box in the white target box for 12 seconds?

0 1 2 3 4 5 6 7 8 9 10

(Not confident at all) (Extremely confident)
