# Supplementary figures and images for: Different Patterns of Neural Activity Characterize Motor Skill Performance During Acquisition and Retention
Source: Front Hum Neurosci. 2022 Jun 13;16:900405. doi: 10.3389/fnhum.2022.900405 (PMC9234574; doi:10.3389/fnhum.2022.900405)

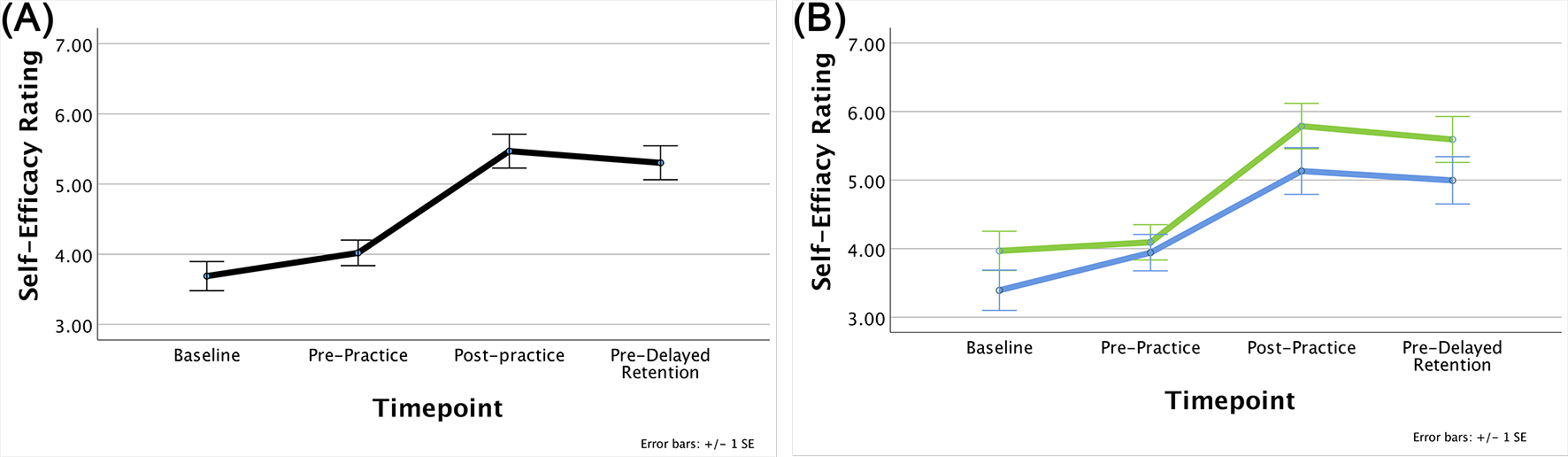

Supplement: Supplementary Figure 1 — Self-Efficacy rating across time points for (A) all participants (N = 40) and (B) motivation plus feedback (green) and feedback only (blue) groups. [file Image_1.TIF]

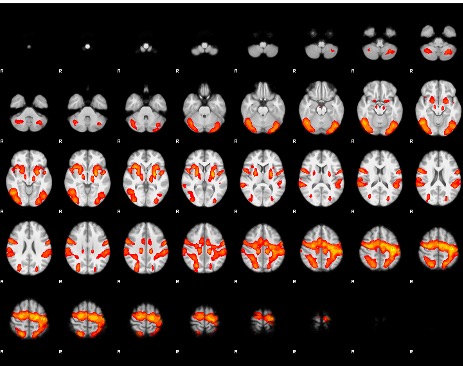

Supplement: Supplementary Figure 2 — Slice-by-slice whole brain activation from Task-related activity analysis. [file Image_2.JPEG]

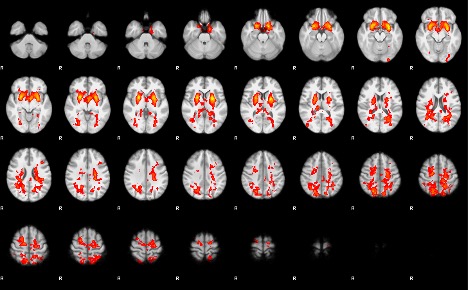

Supplement: Supplementary Figure 3 — Slice-by-slice whole brain activation from Performance specific task activity analysis. [file Image_3.JPEG]

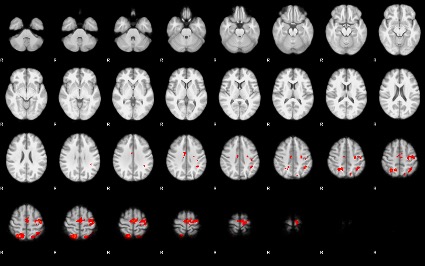

Supplement: Supplementary Figure 4 — Slice-by-slice whole brain activation during practice that is associated with immediate retention performance. [file Image_4.JPEG]

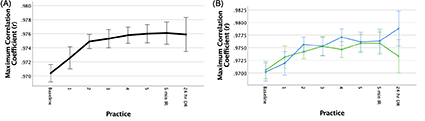

Supplement: Supplementary Figure 5 — Spatial accuracy (maximum cross correlation) during practice and retention for (A) all participants (N = 40) and (B) between groups (motivation plus feedback = green, feedback only = blue). IR, immediate retention; DR, delayed retention. Error bars = ± 1 standard error. [file Image_5.TIF]

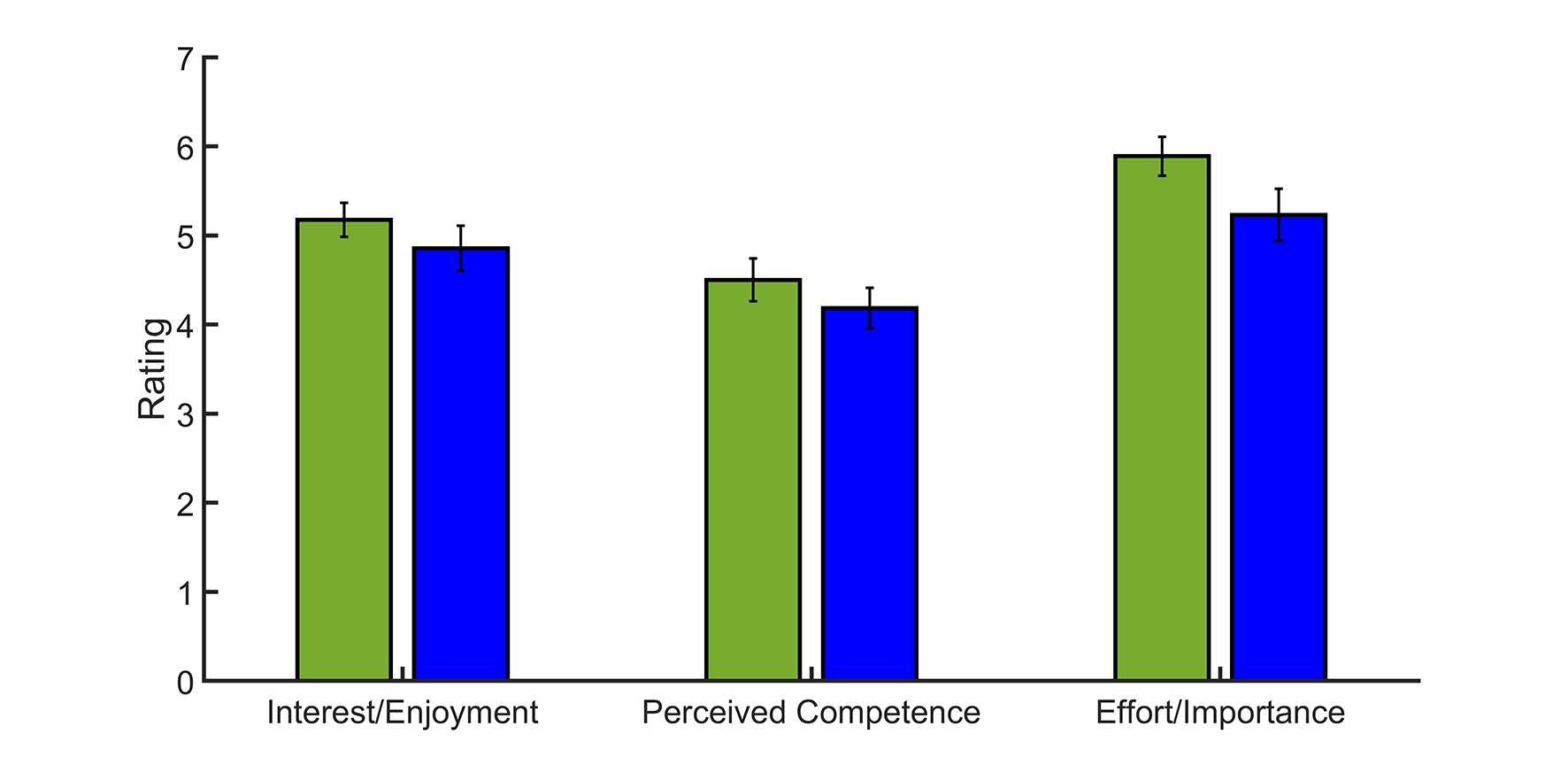

Supplement: Supplementary Figure 6 — Intrinsic motivation inventory subscale rating for motivation plus feedback (green) and feedback only (blue). [file Image_6.TIF]
